# Vertebral Stenting and Vertebroplasty Guided by an Angiographic 3D Rotational Unit

**DOI:** 10.1155/2015/260240

**Published:** 2015-02-23

**Authors:** Escobar-de la Garma Víctor Hugo, Jorge-Barroso Henry Luis, Padilla-Vázquez Felipe, Balderrama-Bañares Jorge Luis

**Affiliations:** Endovascular Therapy Department, Instituto Nacional de Neurología y Neurocirugía “Manuel Velasco Suarez”, Avenida Insurgentes Sur No. 3877, Colonia La Fama, 14269 Mexico City, DF, Mexico

## Abstract

*Introduction*. Use of interventional imaging systems in minimally invasion procedures such as kyphoplasty and vertebroplasty gives the advantage of high-resolution images, various zoom levels, different working angles, and intraprocedure image processing such as three-dimensional reconstructions to minimize complication rate. Due to the recent technological improvement of rotational angiographic units (RAU) with flat-panel detectors, the useful interventional features of CT have been combined with high-quality fluoroscopy into one single machine. Intraprocedural 3D images offer an alternative way to guide needle insertion and the safe injection of cement to avoid leakages. *Case Report*. We present the case of a 72-year-old female patient with insidious lumbar pain. Computed tomography revealed a wedge-shaped osteoporotic compression fracture of T10 vertebrae, which was treated successfully with the installation of vertebral stenting system and vertebroplasty with methacrylate guided with a rotational interventional imaging system. *Conclusion*. Rotational angiographic technology may provide a suitable place for the realization of high-quality minimally invasive spinal procedures, such as kyphoplasty, vertebroplasty, and vertebral stenting. New software programs available nowadays offer the option to make three-dimensional reconstructions with no need of CT scans with the same degree of specificity.

## 1. Introduction

Pain related to vertebral fractures constitutes a more frequent pathologic entity secondary to increases in life expectancy in general population. The number of causes of vertebral fractures is broad, ranging from degenerative, infectious, tumoral, and traumatic fractures [1].

Percutaneous vertebroplasty and kyphoplasty are the now vertebral augmentation procedures that have emerged as minimally invasive surgical options to treat painful vertebral compression fractures during the last 2 decades [2].

Rotational acquisition is a useful supplementary tool to classic PVP and may contribute to patient safety. It is reported that the radiation exposure to the patient with a single rotational acquisition seems to be reduced compared to a standard CT study with quality of images similar to CT scans [3].

## 2. Case Report

**Figure 1:**
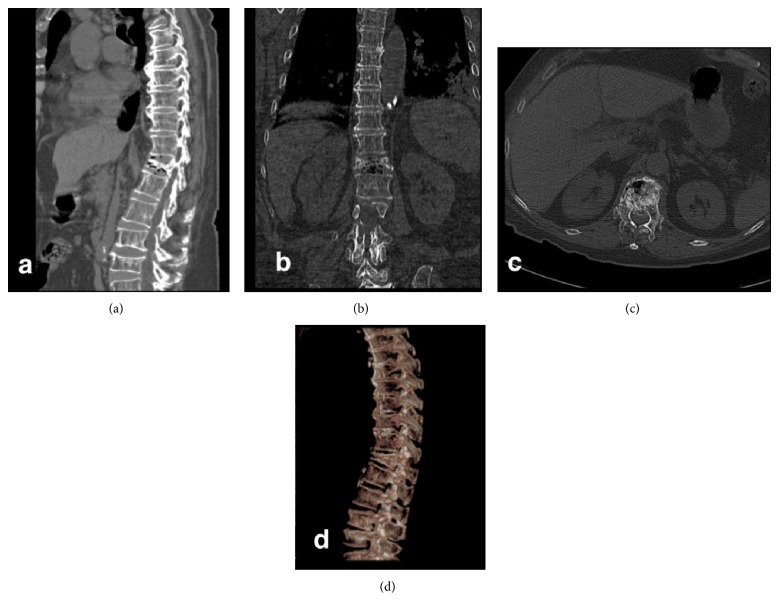
Computed tomography of thoracic spine in (a) sagittal, (b) coronal, and (c) axial projections revealed a T10 vertebral osteoporotic compression fracture and generalized spondylosis of thoracic and lumbar spine. Normal thoracic curvature was affected. (d) 3-dimensional images revealed in detail the wedge-shaped thoracic vertebrae and degenerative changes.

**Figure 2:**
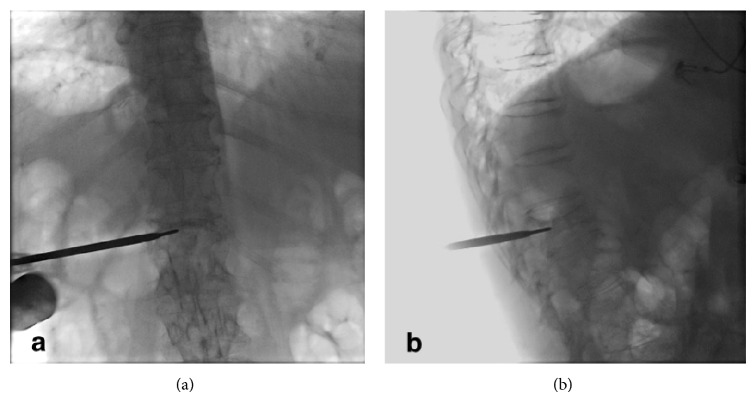
Orthogonal fluoroscopic projections allowed adequate detection of vertebral pedicles of T10 vertebrae for placement of kyphoplasty and vertebroplasty systems.

**Figure 3:**
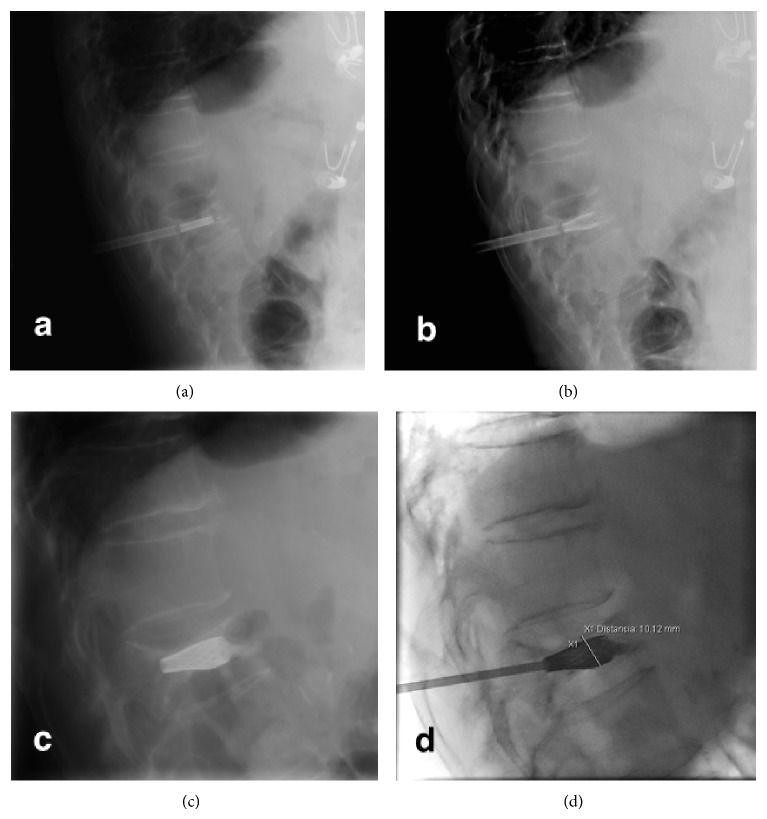
Lateral fluoroscopic projections showed progressive deployment of vertebral body stent with vertebral size augmentation up to 1 cm.

**Figure 4:**
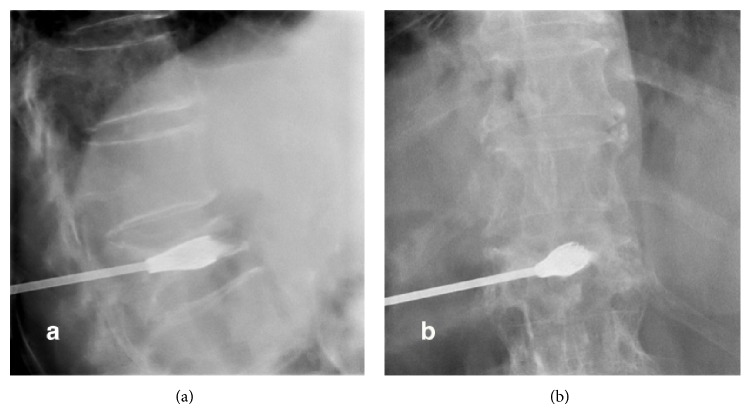
AP and lateral ((a) and (b)) fluoroscopic projections showed the placement of intravertebral cement inside the device previously deployed.

A 72-year-female patient with history of controlled systemic hypertension and osteoporosis was admitted in our hospital. She presented a 2-year history of moderate to severe continuous thoracic and lumbar pain, which was exacerbated with axial movements of the spine. The pain was irradiated to the posterior aspect of the left leg; however, neurologic examination was normal, and neither hypoesthesia nor radiculopathy was found. Computed tomography of the spine revealed a T10 vertebral fracture with a 70–80% of reduction of vertebral height (Figure 1). A minimally invasive spinal procedure was preferred in this case because of the age of the patient. Under fluoroscopic evaluation, a vertebral body remodelling procedure was performed with placement of vertebral body stenting system (DePuy Synthes Spine, Raynham, MA) and subsequent instillation of polymethyl methacrylate guided by a high-quality angiographic unit (Artis Zeego, Siemens, Erlangen, Germany) (Figures 2, 3, and 4). Complementary studies were negative for systemic disease. Vertebral height improved from initial 6.09 mm to 11 mm after treatment. After treatment, lumbar pain was attenuated considerably, with no discomfort to date.

High spatial and contrast resolution of fluoroscopy always provided accurate control of the subsequent injection of polymethyl methacrylate with a neat delineation of the implant margins in case of homogeneous and compact distribution and a detailed visualization of the cement distribution. Acquisition of the images was made and processed with the following technical data: 2k imaging chain; 154 *μ*m native pixel; 30 Å~40-cm rotational flat detector with rotation at 90° in <3 s; detective quantum efficiency (DQE) >65%; fluoroscopy care dose with flexible pulse rates of 10 p/s. (CAREvision).

Rotational acquisitions were always done with patients in the prone position with breath holding for a 6 s time period. A dedicated image protocol (DynaCT-8sDR) was used with a low radiation dose as compared to the traditional angiographic rotational acquisition in order to optimize bone visualization. C-arm rotation (180°–360 images) was done at a frame rate of 60 images/s with a scan time of 8 s.

**Figure 5:**
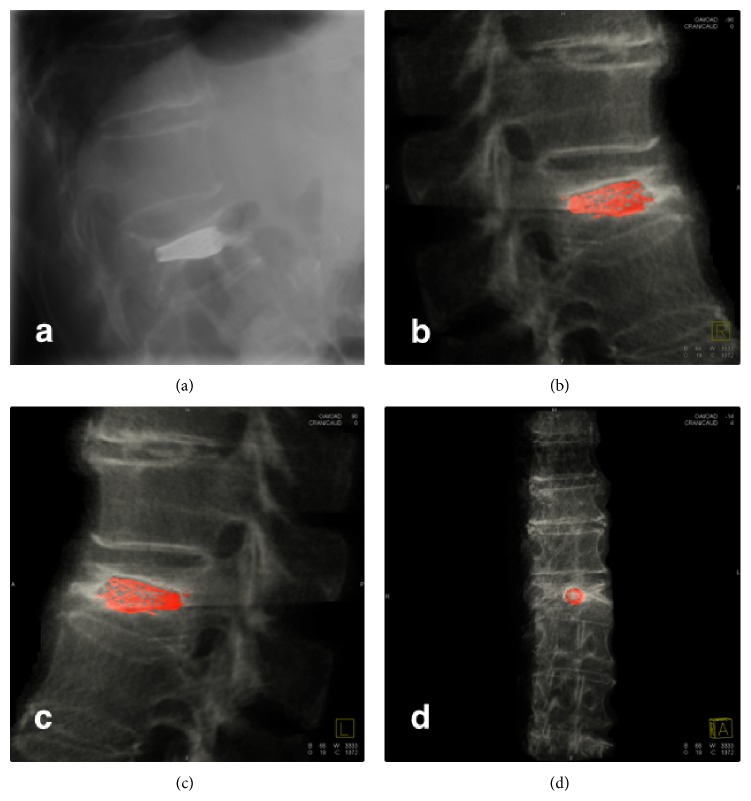
Dual-volume 3D reconstructions in sagittal and coronal projections ((b), (c), and (d)) allowed the separate recognition of prostheses and vertebrae in all angles. Different magnification of the processed images can be done.

**Figure 6:**
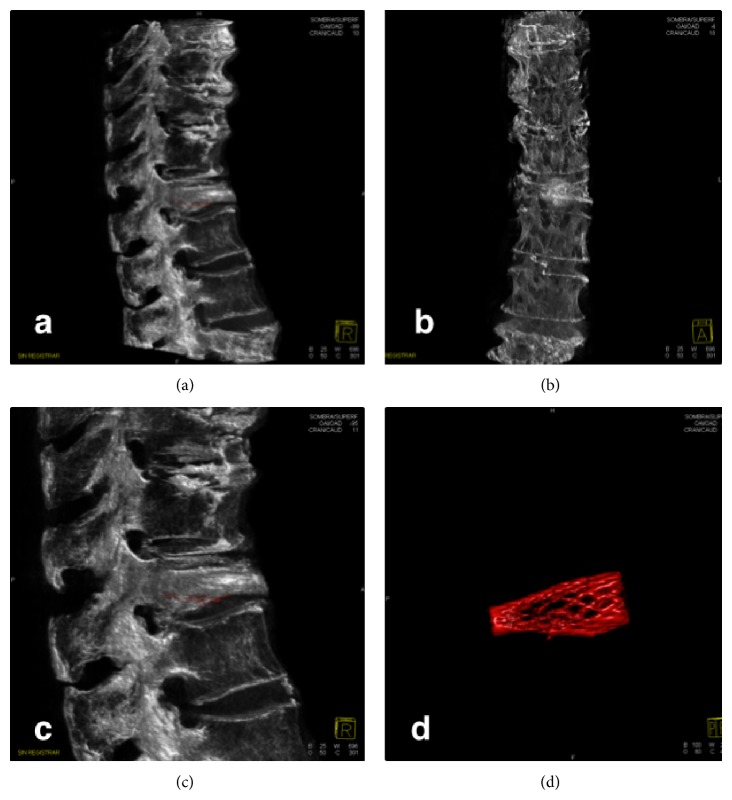
Flat panel detector computed tomography allowed visualization of the disposal of cement inside the affected vertebrae in all angles ((a), (b), and (c)) and the detailed characterization of the intravertebral device (d).

**Figure 7:**
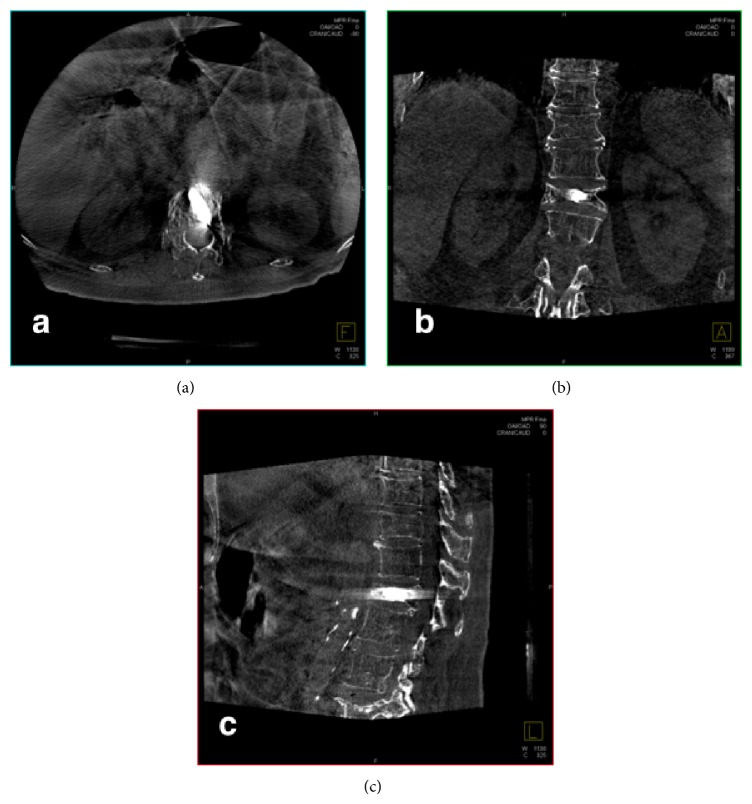
Flat panel detector-CT native images allow CT-like visualization of the disposal of intravertebral cement and its relation to neural foramina and the spinal canal ((a) and (c)).

The acquired images were then transferred to a dedicated workstation (Leonardo workstation, Siemens, Erlangen, Germany) in about 7 s for postprocessing evaluation. Automatic image reconstruction was performed with InSpace 3D software using different filter algorithms for beam hardening, scattered radiation, truncated projections, and ring artifacts. Post-processing resulted in volume data sets with a 512 Å~512 pixel matrix per slice by using different 2D (multiplanar Reformat, MPR) and 3D (volume rendering, VR, or maximum intensity projection, MIP) algorithms (Figures 5, 6, and 7).

## 3. Discussion

Vertebral fracture may result in acute pain around the fracture site, loss of vertebral height due to vertebral collapse, spinal instability, and kyphotic deformity [4]. Typically, the patients with mobile fractures experience pain during coughing, breathing, sneezing, or bending. The pain is mainly related to the motion of the end plate and the micromotion of the trabecular fractures (both of these conditions are the most common histologic findings in osteoporotic fractures) [4].

Vertebral compression fractures can also lead to spinal deformity that may be associated with impaired mobility and physical functioning, decreased pulmonary function, and gastrointestinal problems. These conditions may have a significant impact on quality of life and may even contribute to a reduced life expectancy [1].

There are 3 outcomes in vertebral body augmentation interventions: (a) rapid pain relief, (b) improved body functioning, and (c) vertebral height gain or improved spinal alignment. Thus, the immediate pain relief after vertebral augmentation procedures can easily be related to the cessation of the cleft motion after placement of the bone cement [2].

Percutaneous vertebroplasty and percutaneous kyphoplasty both are effective in vertebral augmentation and pain relief in patients with osteoporotic or tumor-associated vertebral compression fractures. Both procedures have been proven to be superior to oral pain management [2, 4].

The basic procedure (vertebroplasty) involves percutaneous injection of bone cement into the cancellous or spongy bone of the vertebral body to alleviate pain associated with compression fractures, prevent further loss of vertebral height, and correct kyphotic deformity [4]. Kyphoplasty is only a modification of vertebroplasty, and it involves insertion of a balloon into the fractured site, followed by inflation-deflation to create a cavity into which the filler material is injected, and the balloon is taken out prior to cement injection [4]. Vertebral stenting involves the placement of a metallic vertebral prostheses that, after deployment, it is filled with conventional cement [5].

There are some perioperative and postoperative adverse events associated with both vertebroplasty and kyphoplasty, such as symptomatic cement leakage, cement embolism, pulmonary embolism, hematoma, spinal cord compression, radiculopathy, infection, and adjacent vertebral fracture [4]. The overall rate of complications with both procedures ranges from <2% (when treating osteoporotic fractures) to 10% (when treating malignant tumors). Extravasation of bone cement into epidural spaces leads to more serious complications. As a result of bone cement leakage into the venous channel, lethal conditions such as pulmonary embolisms occur, with rates ranging from 0.6% (for vertebroplasty) to 0.01% (for kyphoplasty) [4].

Percutaneous vertebroplasty is characterized by a very low complication rate in the majority of reports, but a small number of serious complications are described and all authors agree about the fact that such events can be minimized by using appropriate high-quality guiding systems [3, 6–8].

Multiple views are required since anterior, posterior, and intradiscal leakages are detectable on lateral views, whereas the frontal view more easily detects leakages in segmental veins often missed on the lateral view [9]. Nowadays, the rotational angiographic unit is considered a valid tool in daily vascular interventional procedures after considerable improvements achieved by technical developments and since its first applications in the seventies. Most of all, it is used for interventional neuroradiology as a useful technique for planning intracranial aneurysms embolization, providing 3D models which can be analyzed by multiple views. Initial reports about 3D RA application for extravascular interventions have also been published [3, 6, 7, 9, 10].

Different studies have shown that angiographic units are reliable and safe when used as the only technique for guidance, control, and postprocedural assessment of vertebroplasty. Avoiding the employment of different imaging modalities can provide a reduction of time and costs. High-quality fluoroscopy as a guiding and control system reduces the risks related to needle access and cement injection. Detection of cement leakage with a rotational image acquisition achieved a good specificity–sensitivity compared to computed tomography, regarded as the gold standard [3, 9].

After the fluoroscopy-guided injection of PMMA, postprocedural assessment is often needed for a precise evaluation of implant distribution. Such assessment is particularly important for the detection of extrasomatic cement leakages especially when they occur in the spinal canal or vertebral foramen. Computed tomography is considered as the method of choice for exact final assessment since fluoroscopic images could produce equivocal findings. Rotational acquisitions with angiographic units have been compared to computed tomography images and are considered of good specificity and sensitivity in the detection of cement leakages and in the assessment of normal anatomical structures and the presence and degree of artifacts [3, 9, 11].

Some studies have shown that rotational angiographic units have the potential to reduce the procedure time and the mean patient dose compared to tomography guided vertebroplasty [3, 10]. Rotational images seem to be of higher quality compared to tomography images and allow safe and fast needle placement, but angiographic images are more sensible for beam hardening artifacts [9].

Some key points should be mentioned: integration of MPR and 3D MIP images provides the best information. In fact, angiographic 2D reformatted images are similar to tomographic slabs and MIP images among other 3D methods allow the best cement visualization and definition, resulting in a better distinction between PMMA and bone structures [9]. The main advantage of rotational angiographic units with respect to traditional angiographic equipments is obviously the possibility of performing therapy control immediately after the cement injection in the same angiosuite with reconstructed rotational acquisitions avoiding the additional charges of a tomographic examination. Rotational acquisition is a useful supplementary tool to classic vertebroplasty and other vertebral augmentation procedures and may contribute to patient safety [3, 6, 10].

With the introduction of rotational image acquisition, it became possible to acquire high-resolution angiographic three-dimensional (3D) image. However, it is possible to employ this technique to other than vascular areas. The feasibility of obtaining volumetric images immediately after an intervention in the angiosuite is clearly the key advantage of rotational acquisitions in comparison to computed tomography imaging [3, 7].

The rotational angiographic technology combines real-time fluoroscopy with a rapid data acquisition of 2D and 3D reconstruction imaging with a high-quality resolution, permitting control in multiple viewing planes of the precise introduction of percutaneous instrumentation and distribution of contrast agents or radiopaque materials, thus minimizing risks and complications [9, 10].

## 4. Conclusion

Rotational angiographic technology may provide the operator with an effective all-in-one technology, and, because of its advanced technical features, it can guarantee safe and efficient guidance for minimally invasive spinal procedures with high-quality three-dimensional images similar to those found in conventional tomographic scans. With the present work, we considered evaluating the reliability of the rotational angiographic unit with flat-panel detector as a single technique to guide vertebral augmentation procedures and for intraprocedural assessment by three-dimensional reformatted images after reconstruction from rotational acquisitions to avoid any potential complication related to the injection of the cement.
